# Prevalence and concentration of *Campylobacter* in faeces of dairy cows: A systematic review and meta-analysis

**DOI:** 10.1371/journal.pone.0276018

**Published:** 2022-10-14

**Authors:** Anna-Delia Knipper, Narges Ghoreishi, Tasja Crease

**Affiliations:** 1 German Federal Institute for Risk Assessment (BfR), Department Biological Safety, Junior Research Group Supply Chain Models, Berlin, Germany; 2 German Federal Institute for Risk Assessment (BfR), Department Exposure, Unit of Epidemiology, Statistics and Exposure Modelling, Berlin, Germany; Beni Suef University Faculty of Veterinary Medicine, EGYPT

## Abstract

The consumption of raw milk from dairy cows has caused multiple food-borne outbreaks of campylobacteriosis in the European Union (EU) since 2011. Cross-contamination of raw milk through faeces is an important vehicle for transmission of *Campylobacter* to consumers. This systematic review and meta-analysis, aimed to summarize data on the prevalence and concentration of *Campylobacter* in faeces of dairy cows. Suitable scientific articles published up to July 2021 were identified through a systematic literature search and subjected to screening and quality assessment. Fifty-three out of 1338 identified studies were eligible for data extraction and 44 were further eligible for meta-analysis. The pooled prevalence was calculated in two different meta-analytic models: a simple model based on one average prevalence estimate per study and a multilevel meta-analytic model that included all prevalence outcomes reported in each study (including different subgroups of e.g. health status and age of dairy cows). The results of the two models were significantly different with a pooled prevalence estimate of 29%, 95% CI [23–36%] and 51%, 95% CI [44–57%], respectively. The effect of sub-groups on prevalence were analyzed with a multilevel mixed-effect model which showed a significant effect of the faecal collection methods and *Campylobacter* species on the prevalence. A meta-analysis on concentration data could not be performed due to the limited availability of data. This systematic review highlights important data gaps and limitations in current studies and variation of prevalence outcomes between available studies. The included studies used a variety of methods for sampling, data collection and analysis of *Campylobacter* that added uncertainty to the pooled prevalence estimates. Nevertheless, the performed meta-analysis improved our understanding of *Campylobacter* prevalence in faeces of dairy cows and is considered a valuable basis for the further development of quantitative microbiological risk assessment models for *Campylobacter* in (raw) milk and food products thereof.

## Introduction

Since 2005 campylobacteriosis, caused by bacteria of the genus *Campylobacter*, is the most commonly reported foodborne gastrointestinal infection in humans in the EU [1]. The EU Member States reported an overall incidence of 120,946 confirmed cases of human campylobacteriosis, corresponding to an EU notification rate of 40.3 per 100,000 population in 2020. Although a decrease in cases was observed in 2020, the overall campylobacteriosis trend in the last four years was stable [1]. Campylobacteriosis symptoms include fever, vomiting, abdominal cramps and watery or bloody diarrhea. Associated chronic complications involve Guillain-Barré syndrome, irritable bowel syndrome and reactive arthritis [2].

Important animal reservoirs for *Campylobacter* spp. are poultry, in particular chicken, and cattle [3, 4]. However, the bacterium is mainly transmitted through contaminated food, direct contact with animals or untreated water [4–6]. In addition to uncooked poultry meat or poor kitchen hygiene in connection with the handling of raw meat, *Campylobacter* infections are frequently reported in connection with the consumption of raw milk and products thereof [1, 7–10]. From 2011 to 2020 raw milk was one of the food vehicles causing most strong-evidence foodborne campylobacteriosis outbreaks in the EU [1]. This is critical in light of the increasing consumer demand for raw milk [11], the intensification of local sales via raw milk vending machines [12] and the common neglect to boil raw milk before consumption. Surveys in Italy demonstrated that 13.9 to 43% of consumers did not boil raw milk before consumption [13, 14].

It is generally assumed that contamination of raw milk with pathogens is mainly of faecal origin [9, 15–17]. However, it is unclear which mechanisms underlie this contamination and how likely raw milk is to be contaminated during milking [14, 15, 18, 19]. In addition, it is also unclear whether there are seasonal differences in the occurrence and concentrations of *Campylobacter* spp. in faeces of dairy cows, which could potentially help to explain the seasonal trend in campylobacteriosis cases [1]. Different mitigation options along the raw milk supply chain need to be assessed in order to understand the role of faecal contamination and a potential seasonality in the public health risk associated with the consumption of *Campylobacter*-contaminated raw milk. Prevalence and concentration data for *Campylobacter* spp. in faeces form a basis for such a risk assessment.

In microbiology, a risk assessment is the qualitative and/or quantitative evaluation of the adverse effects linked to biological agents that may be present in foods [20]. During a quantitative microbial risk assessment (QMRA) the risk is estimated in terms of numerical outcomes, typically the probability of illness or death [21]. Quantitative data, like the concentration in contamination sources (e.g. faeces) or the food matrix, is needed during exposure assessment for the relation between the dose ingested and the frequency of a given effect. To reduce the risk of human exposure to *Campylobacter* spp. it is essential to assess the prevalence and concentration of *Campylobacter* in faeces of dairy cows’. In this sense, a systematic review is necessary to identify all literature on this particular topic. Further a meta-analysis is a highly valuable statistical tool whose objective is to combine the results of all studies on a particular research question to determine the size and direction of the effect.

This systematic review and meta-analysis aimed to provide and estimate the prevalence and concentration of *Campylobacter* in dairy cow faeces. Moreover, potential data gaps for risk assessments were identified in order to highlight where further research is needed. The knowledge and data generated from this study is ought to contribute to the development of QMRAs and the evaluation of different contamination or exposure scenarios along the raw milk supply chain, thereby helping risk managers to identify mitigation strategies to control *Campylobacter* spp. and to reduce the public health risk associated with the consumption of *Campylobacter*-contaminated raw milk.

## Material and methods

### Literature search and inclusion criteria

A systematic review was performed according to the Preferred Reporting Items for Systematic Review and Meta-Analysis Protocols (PRISMA-P) 2015 statement [22] (S1 Checklist). A pre-specified study protocol was published on the International Prospective Register of Systematic Reviews (PROSPERO) database (CRD42021261914, https://www.crd.york.ac.uk/prospero/display_record.php?RecordID=261914), in order to avoid duplication and to minimize bias. Literature searches were carried out using PubMed, Scopus and Web of Science databases for papers published to July 19th 2021. A detailed overview of search terms per database is provided in Table 1. Synonyms for relevant search terms were identified using the Medical Subject Headings (MeSH) thesaurus by the US National Library of Medicine [23] (https://www.nlm.nih.gov/mesh/meshhome.html).

**Table 1:** Overview of search strategy and number of articles found specific to the respective datatbase.

Date Search performed	Database	Number of articles retrived	Search string/terms and limits
19. July 2021	PubMed	453	All = (Search #1) AND All = (Search #2) AND All = (Search #3)
19. July 2021	Scopus	485	Abstract, Title, Keyword = (Search #1) AND Abstract, Title, Keyword = (Search #2) AND Abstract, Title, Keyword = (Search #3)
19. July 2021	Web of Science	400	TOPIC = (Search #1) AND TOPIC = (Search #2) AND TOPIC = (Search #3)
		Where:	
		Search #1	(Campylobacter*)
		Search #2	(cow) OR (cattle) OR (bovine) OR (ruminant) OR (dairy) OR (heifer) OR (calf) OR (bos indicus) OR (zebu) OR (bos grunniens) OR (yak) OR (bos taurus)
		Search #3	(feces) OR (faeces) OR (excrement) OR (fecal) OR (faecal) OR (dung)

A title and abstract screening was performed, followed by a full-text screening for eligibility for inclusion and exclusion criteria already defined in the PROSPERO protocol and for the removal of duplicate publications of the same results or study. If the answer to the *a priori* defined exclusion criteria remained unclear during the initial screening the study was forwarded to the full-text screening. All relevant articles were uploaded to the Rayyan Systems Inc. [24] web tool for efficient organization of inclusion and exclusion and to document the reasons for exclusion. Two researchers (ADK, TC) performed both screenings independently in Rayyan. Discrepancies were resolved by a third researcher (NG). Studies were excluded if they met the pre-defined exclusion criteria.

### Data extraction

Full text articles were examined and relevant data was extracted from text and tables into purpose-built tables using Microsoft Excel 2016 (Microsoft Corp., Redmond, WA, USA). Metadata on the general study design and metadata related to each reported outcomes was extracted separately. The following general metadata was extracted from each study: year of publication, country of study, faecal collection method, method for *Campylobacter* detection/ enumeration and species identification, number of dairy cow farms sampled, age class of cows, health status of cows, whether repeated samplings for individual cows or cow farms were performed, whether the repeated outcomes for individual cows or for cow farms were reported, and whether the available repeated outcome were reported by season (i.e. summer, fall, winter, spring).

Each study may comprise more than one prevalence outcome e.g. derived from different sub-groups or sampling conditions (i.e. *Campylobacter* species, age class, health status, seasons) and outcomes may be reported repeatedly within one study based on different sub-grouping or data aggregation. All relevant prevalence outcomes were extracted and the sub-grouping was documented in the metadata. Each extracted outcome was associated with the following additional metadata: *Campylobacter* species, season, number of faeces samples collected, health status of cows and age class of cows.

The review and data extraction was performed by two researchers (ADK, TC) individually and tables were subsequently merged. Discrepancies were resolved by discussion or consultation of a third researcher (NG). Authors of included articles were not contacted in case of missing data. The created database was double-checked independently by two researchers (ADK, TC).

### Bias assessment

There is currently no validated tool for risk of bias (RoB) assessment in observational animal studies including prevalence studies. The available tools are appropriate for animal experiments (e.g. SYRCLE [25], CAMARADES [26]) or human observational studies (e.g. ROBINS-I [27]). As a result, risk of bias was assessed based on a purpose-built modified RoB tool. Applicable questions from the above mentioned tools were gathered in a table and adapted for prevalence studies (e.g. were rephrased or split into multiple, more study specific criteria). In total ten questions were included in the final tool (S2 Table). During the data extraction, the reviewers also filled the RoB tool for each study, counted the number of “yes”, “no” and “unclear” answered questions, and labeled studies with more than four “yes” answers as “low risk of bias”. Questions answered with “no” and “unclear” contributed to high risk of bias. No funnel plot was drawn since funnel plots are not appropriate for assessing the publication bias in studies with prevalence outcomes [28].

### Description of data sets for meta-analysis

Study outcomes for pooled faecal samples and outcomes where the number of animals sampled was unclear or not specified were excluded from the meta-analysis. As described in section data extraction, we extracted all relevant prevalence outcomes from each study. This introduced duplications of the same data under different sub-groupings (i.e. *Campylobacter* species, age class, health status, and seasons) in our data set for meta-analysis. To consider the effect of these duplicates on the analysis we chose to work with two different data sets and meta- analytic models. One dataset was reduced to only those prevalence outcomes that were reported as an average across the whole study (e.g. across all potential sub-groups such as age class, health status and seasons). This dataset will hereafter be referred to as aggregated sample (S1 Table). The other dataset included all extracted outcomes, including potential duplications due to different sub-groupings and data aggregation. The method for meta-analytic model was chosen accordingly. This data set will hereafter be referred to as the non-aggregated sample (S1 Table).

Potential influencing factors of interest were, the season during faecal sample collection, the *Campylobacter* species, the age class and the health status of the cows as well as the faecal collection method. The effect of these factors on the prevalence estimates was further investigated via statistical analysis.

### Statistical analysis

We used R Software version 4.1 for statistical analysis [29] and the packages “meta” [30] and “metafor” [31] for the development of the meta-analytic models.

#### Meta-analytic models

Two meta-analytic models were used to estimate the pooled prevalence. In the first model the prevalence outcomes of the aggregated sample were included in a random effect model for proportions with an inverse variance method, which we will refer to as simple model.

In the second model, the non-aggregated sample was included in a multilevel model where prevalence outcomes reported in each study were in one level and studies were compared in the other level. For each level an inconsistency index (I^2^) was calculated as a measure of heterogeneity which is defined as the percentage of variability in the effect estimates that is not explained by the sampling error. In both models the estimates were double arcsin transformed.

#### Subgroup analysis

For subgroup analysis, we used the aggregated sample prevalence if at least three outcomes from different studies were available. The Q-test was used to test the difference between the subgroups.

#### Effect of subgroups on the prevalence

We performed an analysis on the non-aggregated sample (using all the extracted outcomes) to investigate the effect of subgroups on the pooled prevalence estimate based on a multilevel mixed-effect model with restricted maximum-likelihood estimation (REML). The model features included the *Campylobacter* species, health status and age class of the dairy cows, the season of outcome measurement and the faecal collection method. As with the previous multilevel model, the prevalence outcomes reported for each study were considered as one level and the comparison between the studies was calculated in the other level.

#### Meta-regression

We performed a meta-regression to evaluate the effect of the publication year of studies on the prevalence estimates. For this analysis, we added the publication year as a variable to the simple model regression and created a graph of the prevalence values versus publication year.

#### Sensitivity analysis

The created data table for RoB analysis was used to estimate the pooled prevalence for the high and low risk of bias studies of the aggregated sample and the results were compared using a Q-test. As the second sensitivity analysis, the pooled prevalence estimate from the aggregated sample in the simple model and results from the pooled non-aggregated sample in the multilevel model were compared.

## Results

### Search summary of the systematic review

**Fig 1:**
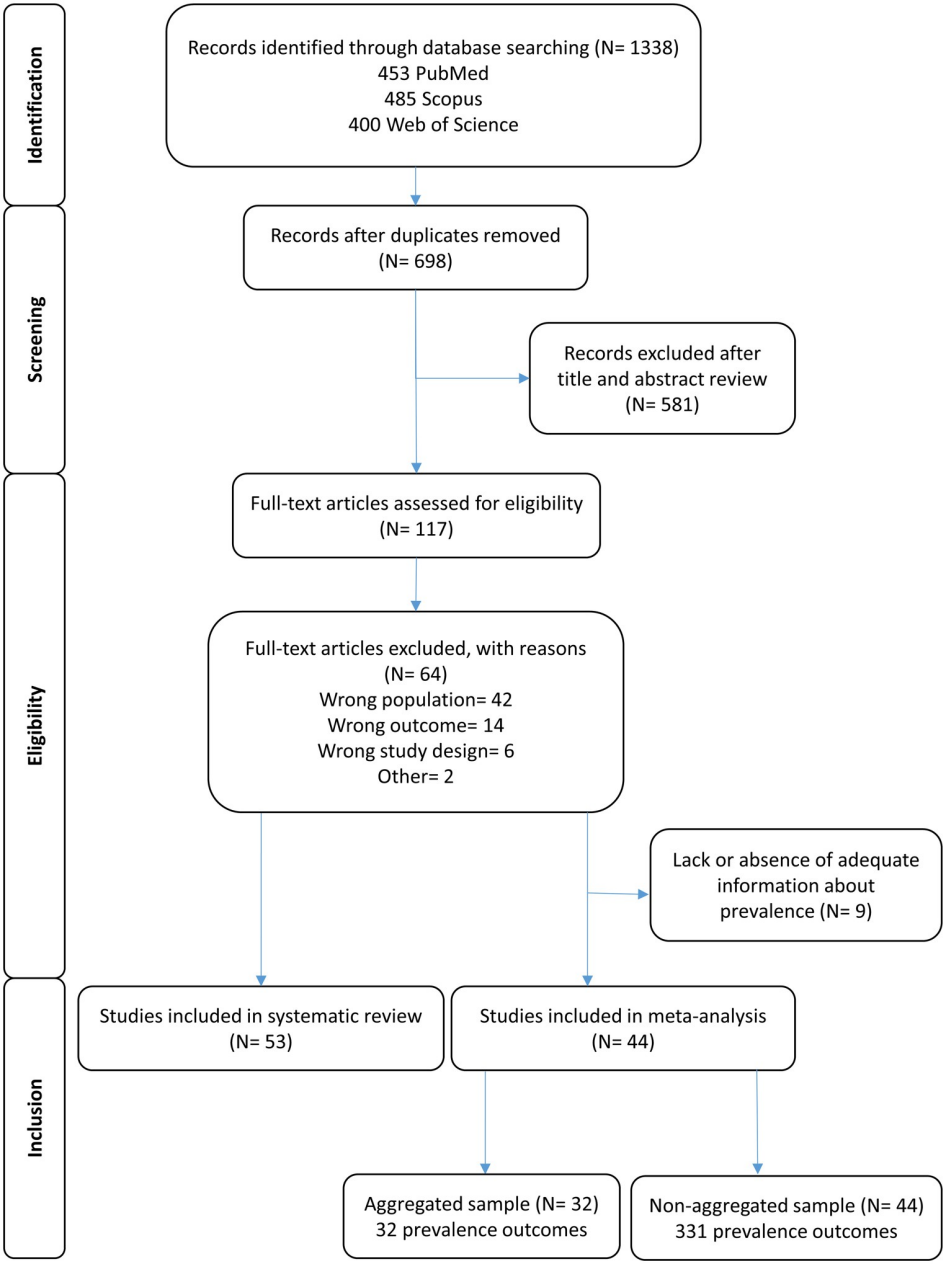
Flow diagram of selected studies included in the systematic review and meta-analysis. Aggregated sample means a specific prevalence outcome was reported as an average outcome across the whole study, whereas with non-aggregated sample an outcome was reported for a specific sub-group or condition.

Fifty-three out of 1338 identified studies were eligible for data extraction after screening and eligibility testing according to PRISMA-P (Fig 1).

Of these, 17 studies were from Europe (32%), 15 from North-America (28.3%), seven from Oceania (13.2%), six from Asia (11.3%), five from South-America (9.5%) and three from Africa (5.7%). Most of the Europe-based studies were from the UK (N = 5; 9.4%). Other European countries i.e. Austria, Denmark, Germany, Latvia, Lithuania and Sweden were represented by one study each, while Finland, Italy and Sweden were represented by two studies.

**Fig 2:**
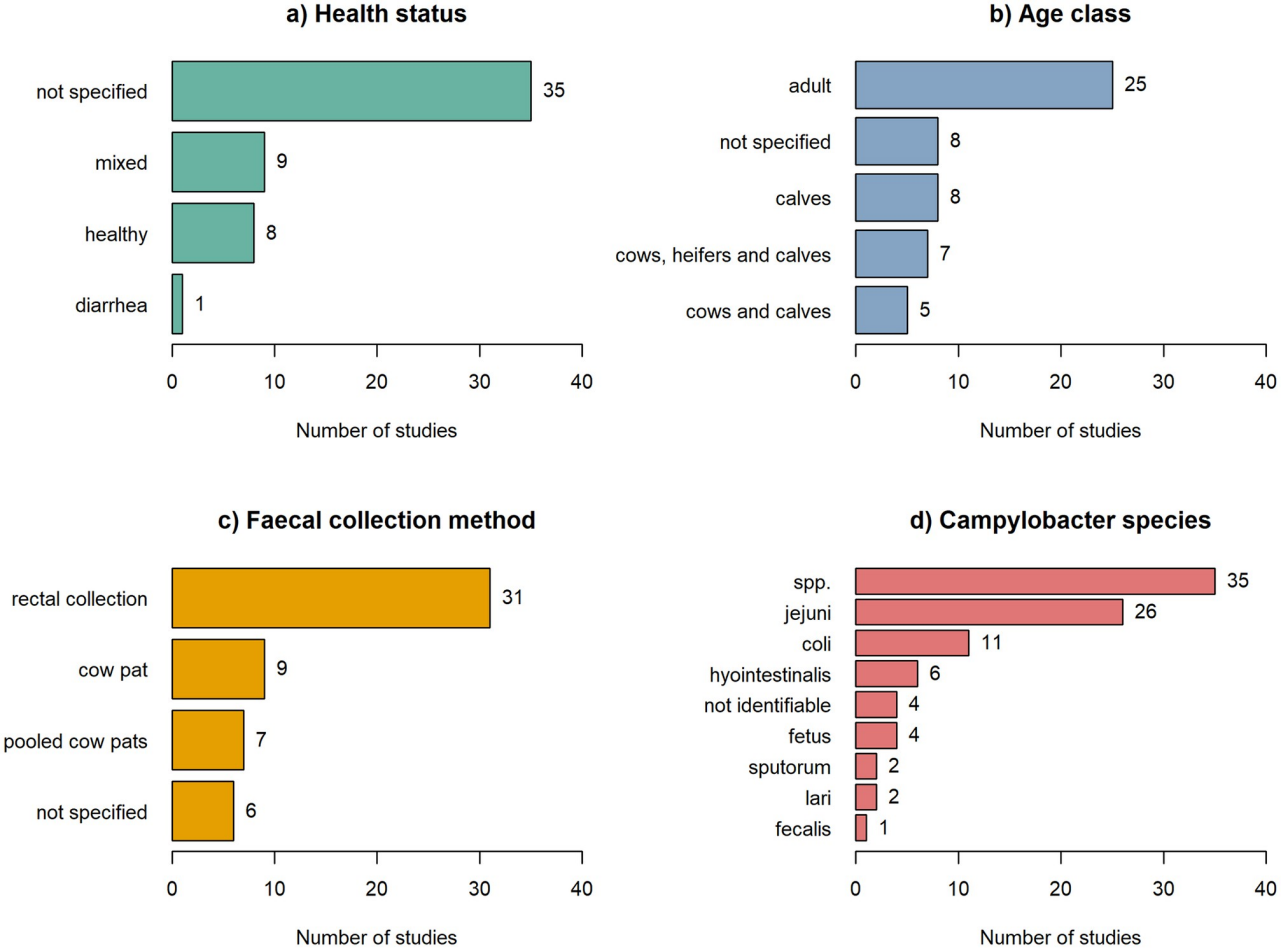
Number of studies reporting data for potential influencing factors and their subgroups, which are the health status (a) and age class (b) of dairy cows, as well as the faecal collection method (c) and the *Campylobacter* species (d). In some studies the collected faecal samples were analyzes for more than one species.

On average, 432 (± 678) dairy cows and 21 (± 34) farms were sampled in the included studies. The health status of the sampled dairy cows was not specified in a majority of studies (N = 35; 66%), while other studies (N = 18; 34%) gave a clear description of the health status of the dairy cattle (Fig 2a). Different age groups of dairy cows were sampled throughout the included studies (Fig 2b). However, in some studies no description of the age group of cows was given (N = 8; 15%).

The faecal collection methods were the collection of cow pats from the floor (N = 9; 17%) and direct rectal extraction methods (N = 31; 58.5%). In six studies (11.3%) the faecal collection method was not stated and some studies pooled faecal samples before analysis (N = 7; 13.2%) (Fig 2c). *Campylobacter* was mainly detected by culture-based methods (N = 49; 92.5%). Only a few studies used PCR-based methods (N = 3, 5.7%) or a combination of PCR- and culture-based methods (N = 1; 1.9%). The majority of studies (N = 37; 69.8%) tested faecal samples for two or more *Campylobacter* species (including *Campylobacter* spp.). *Campylobacter* spp. (N = 35; 66%) and the species *C*. *jejuni* (N = 26; 49%) and *C*. *coli* (N = 11; 20.8%) were most commonly reported in all included studies. Other species such as *C*. *hyointestinalis*, *C*. *fetus*, *C*. *sputorum*, *C*. *lari*, and *C*. *fecalis*, were rarely tested for, while some species could not be identified (Fig 2d).

Almost 50% of all studies (N = 26) reported repeated samplings for farms under study. Only some of these (N = 15; 28.3%) were taken according to seasons in temperate regions (i.e. spring, summer, autumn, winter), while others (N = 5; 9.4%) were taken according to rainy and dry season, depending on the geographical location of the country. In general, only few studies (N = 14; 26.4%) made the results of the repeated sampling explicitly available in their publication. This means that although repeated samplings were taken, the results of these samplings were not reported individually, but rather aggregated or not shown at all. Repeated sampling for individual cows were only taken in a small number of studies (N = 5; 9.4%), but none of these studies made the results for individual cattle available in their publication. Data extracted from publications and included in systematic review and meta-analysis are available in S1 Table.

### Risk of bias assessment

The number of “yes”, “no” and “unclear” answers for each RoB criteria is shown in S2 Table. No study answered all the RoB criteria with “yes”. The highest answer rate was eight out of ten “yes” answers for one study. Twenty-two studies (42%) had four or more “yes” answers which was considered as low risk of bias. Results of the meta-analysis on prevalence outcomes of RoB sub-groups are further presented in result section sensitivity analysis.

### Findings from the concentration outcomes

Concentration outcomes were only reported in seven (13.2%) of the 53 studies included in the review. The provided concentration outcomes in three of these studies [32–34] was a semi-quantitative estimate, which was determined by the most probable number (MPN) method for *Campylobacter* spp.. Concentration outcomes from another study could not be extracted as they were only presented in a box plot [35]. A meta-analysis for the remaining three studies [36–38] with quantitative concentration outcomes could not be performed, as one of these studies [38] did not provide any standard deviation or confidence intervals for the reported concentration.

The average *Campylobacter* spp. concentration in Danish dairy farms of 120 dairy cows was 2.1 ± 0.45 log colony-forming unit (CFU)/g faeces [36]. In contrast, a Lithuanian study determined for cows higher concentrations of 3.55 ± 0.92, 4.17 ± 0.54, 3.29 ± 0.44 log CFU/g faeces in three different dairy farms [37]. Another study from the United Kingdom found similar average concentrations with seasonal differences of 1 log CFU/g faeces between summer and winter, with an average of 3.2 log CFU/g faeces in summer and 4.2 log CFU/g faeces in winter [38].

### Findings from the meta-analysis on prevalence outcomes

After excluding the studies with prevalence outcomes reported for pooled faecal samples and studies where the number of dairy cows sampled was not clear, 44 studies remained.

Out of these 44 studies, only 32 studies reported a prevalence for the aggregated sample, which equates to 32 prevalence outcomes. For the non-aggregated sample, including these 44 studies, 331 prevalence outcomes for different sub-groups and conditions were reported (S1 Table).

**Fig 3:**
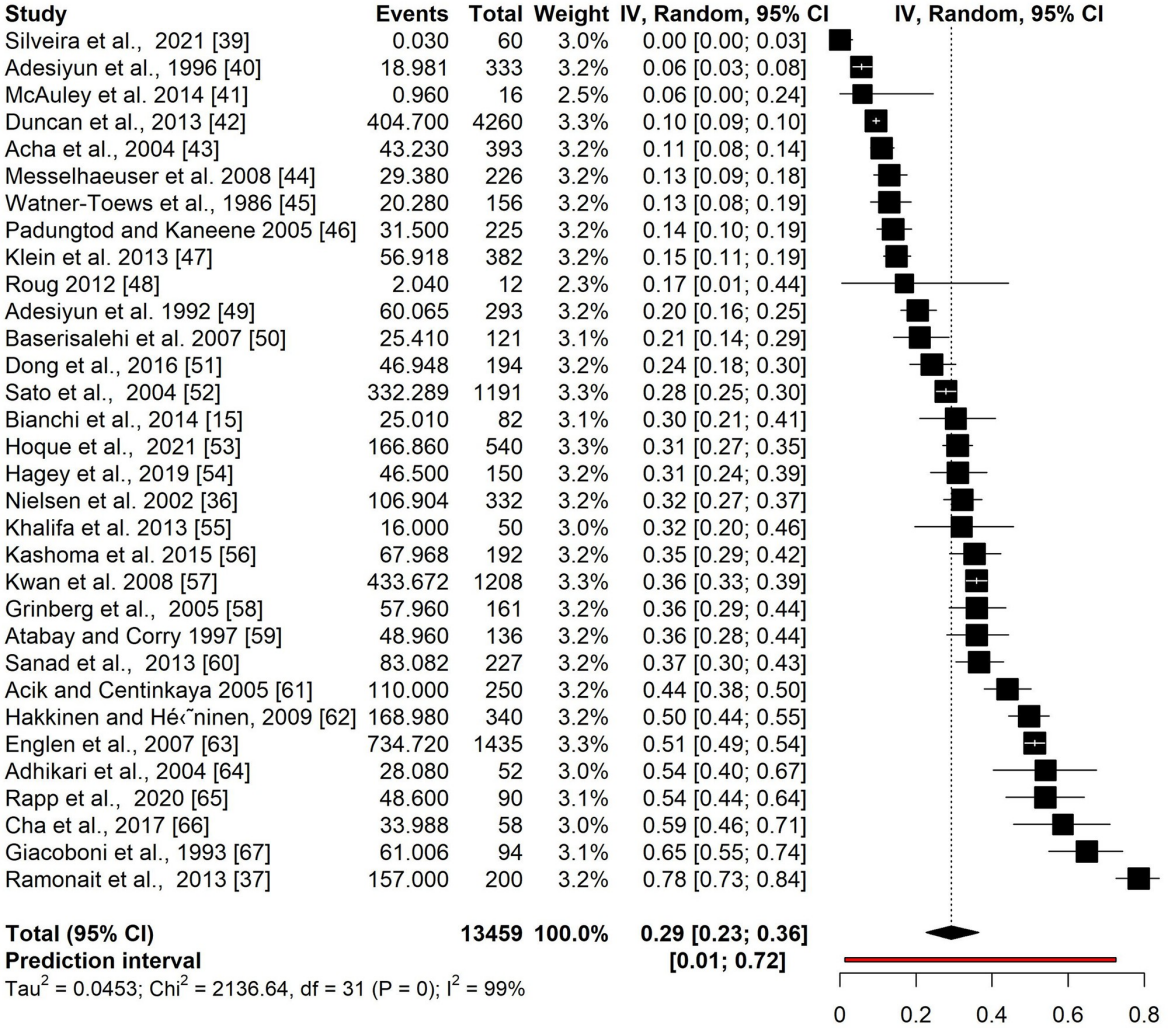
Forest plot of the aggregated sample estimating the pooled prevalence of *Campylobacter* spp. in cows’ faeces from 32 studies. Event is pooled prevalence times number of individual cattle sampled.

The overall prevalence estimate of the simple model that was based on the 32 prevalence outcomes of the aggregated sample was 29.3%, 95% CI [23–37%] with high heterogeneity I^2^ = 98.5% [98–99%] and a prediction interval of 1.3% to 73% (Fig 3) [15, 36, 37, 39–67].

The pooled prevalence estimate of the multilevel model that was based on the 44 eligible studies and all their pooled prevalence was 51% with 95% CI [44–57%] and I^2^ = 97.96% and a prediction interval of 0% to 100%. The sampling error was 2.04%. The heterogeneity within studies was 62.86% and the amount of between study heterogeneity constituted 35.1% of the total variation in our study (S1 Fig).

#### Subgroup analysis

A sub-group analysis of the aggregated sample was performed for the faecal collection method and the age class of cows. All other sub-groups in the aggregated sample could not be analysed because too few prevalence outcomes per group (N<3) were available.

**Fig 4:**
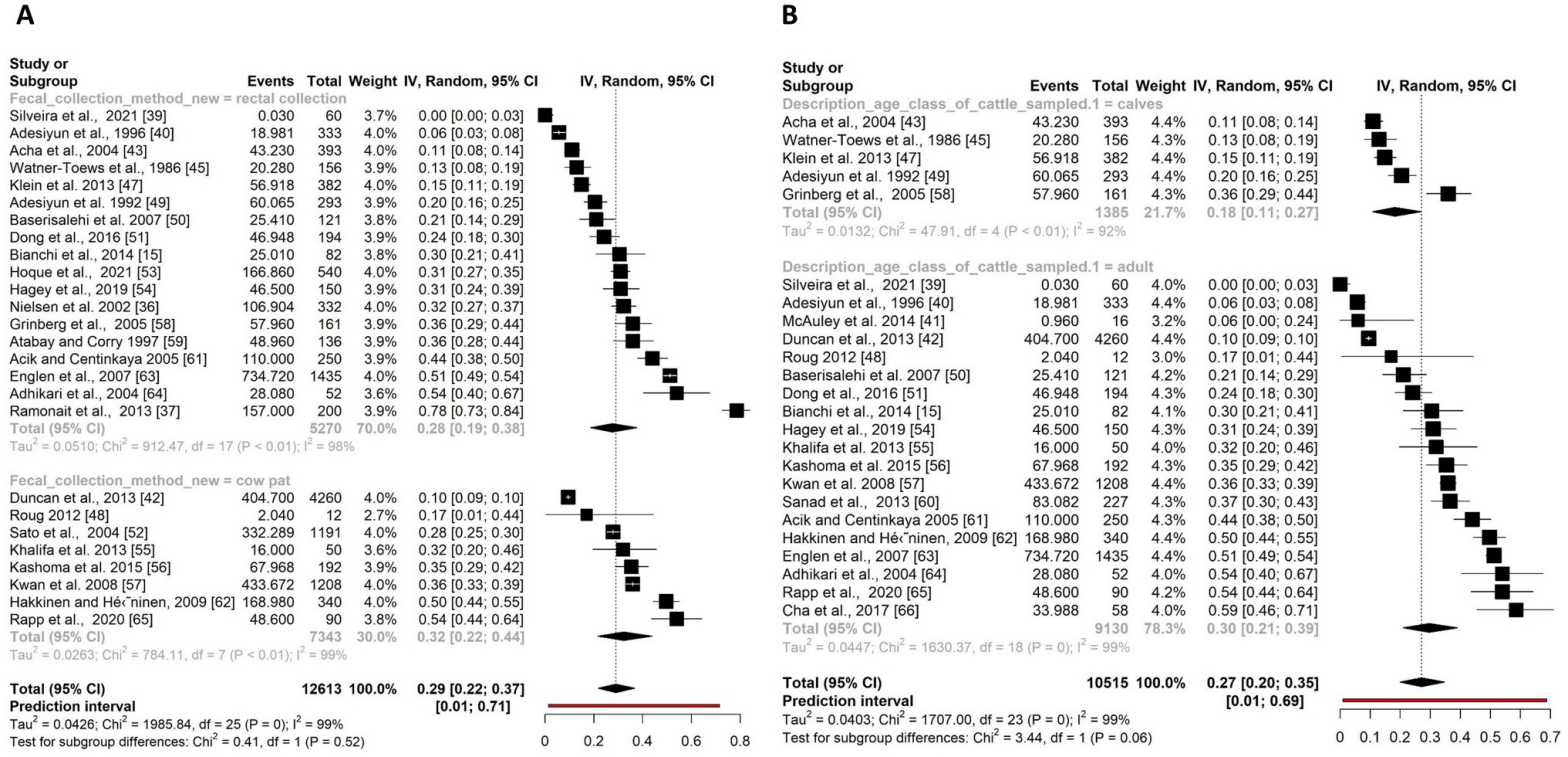
Forest plot of the sub-group analysis of the aggregated sample comparing the prevalence estimates of *Campylobacter* spp. in faeces of dairy cows between different faecal collection methods (A) and age classes of the dairy cows (B). Event is pooled prevalence times number of individual cattle sampled.

For the faecal collection method, the prevalence outcomes between a rectal faecal extraction (18 studies) and the collection of cow pats (eight studies) from the floor (of the stable or meadow) were compared (Fig 4a). The prevalence estimate for the rectal extraction was 28%, 95% CI [19–38%] and for the cow pat collection 32%, 95% CI [22–44%]. The difference between these prevalence estimates was not significantly different (p = 0.52).

Only the prevalence outcomes of calves (five studies) and adult cows (19 studies) could be compared for the aggregated sample. For heifers, not enough aggregated outcomes were available (N<3) to be included in the analysis. The prevalence estimates for calves and adult cows were 18%, 95% CI [11–27%] and 30%, 95% CI [21–39%] respectively (Fig 4b). No significant difference between these results was found (p = 0.06).

#### The effect of subgroups on the prevalence

The multilevel mixed-effect model showed a variance of 3.7%, 95% CI [1.9–7.3%] between studies and a variance of 3.8%, 95% CI [3.1–4.7%] for within study variance estimates. The variables *Campylobacter* species *C*. *hyointestinalis* and *C*. *jejuni* and the rectal faecal collection had a significant impact on the prevalence. The heterogeneity measure within the studies after accounting for the subgroups was 49.46% and the heterogeneity between the studies accounted for 48.29% of the total variability (S1 Fig).

#### Meta-regression

In addition, we assessed the effect of study year of publication on the prevalence estimate in a meta-regression of the aggregated sample. The meta-regression showed that the study year explained less than 1% of the heterogeneity (0.88%) observed in the prevalence outcomes and was not significantly affecting the prevalence estimate. The bubble plot based on the meta-regression confirms the finding (S2 Fig).

#### Sensitivity analysis

The risk of bias assessment was performed on all studies included in the systematic review (N = 53) and 22 (42%) of these grouped as low risk of bias. In the studies included in the simple meta-analytic model 13 of the 32 studies (41%) were in the low risk of bias group. The pooled prevalence estimate in the simple meta-analytic model was 32.5%, 95% CI [22–44%] and 27%, 95% CI [18–37%] in the low and high risk of bias group, respectively (p = 0.45) (S3 Fig). The second sensitivity analysis was the comparison between the pooled prevalence estimate from the simple and multilevel meta-analytic model. The analysis showed a significant difference between the two models. The estimated prevalence was 29% [23–36%] and 51% [44–57%] and the prediction values were [1–73%] and [0–100%] for the simple and multilevel meta-analytic model, respectively.

## Discussion

Based on the increasing consumer demand for fresh and raw products and the resulting consumption of unboiled raw milk, the raw milk supply chain has become more of a focus in recent decades. Especially because raw milk is one of the top vehicles causing strong-evidence outbreaks in the EU [1]. This might have contributed to the increase in studies focused on prevalence of *Campylobacter* in dairy cows faeces in the last 20 years (S2 Fig). In addition, animal health and farm management are further reasons for increased studies. However, systematic reviews and meta-analysis which allow for an estimation of the prevalence and concentration of *Campylobacter* spp. in cow faeces and identify potential data gaps have not been carried out yet. The assumption that *Campylobacter* contamination of raw milk is mainly caused by faecal contamination highlighted the importance of such systematic review and meta-analysis [9, 15–17]. The prevalence and concentration of *Campylobacter* spp. in faeces of dairy cows form an important basis for the mathematical modelling (via QMRAs) of potential cross-contamination events and mechanisms along the raw milk supply chain. The development of such QMRAs can furthermore help to identify different mitigation options along the supply chain in order to reduce the public health risk associated with the consumption of *Campylobacter*-contaminated raw milk.

Here, we could only extract quantitative data on the concentration of *Campylobacter* spp. in faeces of dairy cows from three studies, as other studies gave only semi-quantitative estimates of the concentrations or presented results in a figure, which did not enable the extraction of e.g. a mean and standard deviation for the concentration. The average reported concentration of *Campylobacter* in faeces varied between the three studies and a meta-analysis could not be performed due to missing uncertainty measures (e.g. standard deviations). Specifically concentration data (including mean and standard deviation) are an important input for QMRAs, because the risk is the product of the probability that a random serving is contaminated and the probability that a contaminated serving results in disease. To clarify, the probability that a random serving is contaminated is based on the prevalence data and the probability that a contaminated serving results in diseases are calculated with concentration data that are used as input for the dose-response relationship [20, 21, 68]. These results clearly highlight the lack of concentration data (including uncertainty measures), which currently impedes risk assessments and consequently the refinement of mitigation options to reduce the public health risk from contamination of *Campylobacter* in cows’ faeces.

The prevalence data for *Campylobacter* in dairy cow faeces were widely available in the scientific literatures, however, the range of prevalence varied highly (0–100%). In addition, some of the studies differed greatly in study design and quality e.g. in the specific and often missing information, e.g., on the health status studied (Fig 2). Subgroup analysis could therefore only be performed for the faecal collection method and the age class of dairy cows. All other subgroups of influencing factors of interest (i.e. the season during faecal sample collection, the *Campylobacter* species, and the health status of the cows) could not be compared because less than three prevalence outcomes per group were available.

Our RoB analysis could have been improved using a validated tool for observational animal studies. We hope future studies develop such a tool to make RoB analysis more standardized among prevalence studies. In addition, the RoB analysis showed that less than half of the studies are having a low risk of bias. It also showed that only five studies explicitly mentioned the application of ISO methods for *Campylobacter* detection and characterization. For most studies (N = 42) it remained unclear (meaning that it was not explicitly mentioned) whether an ISO method (e.g. ISO10272-1:2017 [69] and/or ISO10272-2:2017 [70]) was used. A detailed subgroup analysis of studies with and without the application of ISO methods was also not possible due to too few prevalence outcome in each group. This emphasizes the problem of wide heterogeneity between the studies further, especially since the detection and characterization of a sensitive bacterium such as *Campylobacter* spp. has proven challenging [71, 72].

The meta-analytical models aimed to estimate the pooled prevalence and to subsequently evaluate which influencing factors might affect the prevalence estimates and to some part explain the heterogeneity. The multilevel model offered the opportunity to include all extracted prevalence outcomes (N = 331) from the 44 studies. The pooled prevalence estimate from this model was higher than the estimate from the simple model. The prediction interval was also wider going from zero to one, better reflecting the heterogeneity between the outcomes. When adding the subgroups to the multilevel model the results were in some cases different to subgroup analysis based on aggregated sample (e.g. for faecal collection method). For the mixed-effect multilevel model, the variables of *Campylobacter* species *C*. *jejuni* and *C*. *hyointestinalis* (in comparison to *coli*) and rectal faecal collection method (in comparison to cow pat collection) additionally had a significant impact on the pooled prevalence estimate. The subgroup analysis, in contrast, showed no difference in prevalence between the two faecal collection methods (rectal collection and cow pat) probably due to the remaining heterogeneity between the two subgroups, which have been adjusted for to an extent in the multilevel mixed-effect model.

Heterogeneity between studies was also evident in all meta-analytic models and their high inconsistency index (Fig 3 and S1 Fig). The variation was most likely a result of the different study designs and the subgroup differences. In the multilevel model it was evident that the variation between studies contributed less to the total variation than the within study variance. When subgroups were included in the multilevel model the within study variance decreased from 62.86% to 49.46% and as a result the between study variance accounted for almost half of the total variability (from 35.1% to 48.29%). Thus, making an estimation of the prevalence of *Campylobacter* in faeces of dairy cows difficult based on current studies.

Interestingly, mixed-effect multilevel model showed a significant effect of the faecal collection method on the pooled prevalence estimate. However, the subgroup analysis of aggregated samples in this study showed no significant difference between the prevalence obtained by rectal extraction (28%) or cow pats (32%) (Fig 4a). These findings were contrary to a study by Hoar et al., [73] that showed that the prevalence in cow pats was lower compared to rectal extraction in beef cattle. Nevertheless, the prevalence obtained in this study were quite low with only 5% for rectal faecal samples and 0.5% for cow pats [73]. We assumed that the cow pats in most of the studies included in this review and meta-analysis were examined immediately after shedding, which could explain the high prevalence found in cow pats. Another reason could be that the rectal extraction is not necessary allow for a mixture of a large amount of faeces, but rather supports the extraction of a few grams (e.g. rectal swab), which might not reflect the true prevalence. However, these findings also emphasize that *Campylobacter* already exhibits several survival strategies to adapt harsh conditions, e.g. in cow pats, by genetic exchange [74], by adaption mechanisms [75–77] or undergoing the viable but non-culturable state [78]. Accordingly, the survival of *Campylobacter* in cow pats in the stable environment may have been underestimated in the past.

The subgroup analysis of the aggregated prevalence estimates for calves and adult cows were 18% and 30%. The lower prevalence in calves could possibly be due to the use of straw compared to the stalls of adult cows [79]. Anyway, no significant difference between these results was found based on the subgroup analysis (Fig 4b). The multilevel mixed-effect model also showed no significant effect of the subgroups on the pooled prevalence estimate. In the search for quantitative data, two studies were identified that detected significantly higher concentrations of *Campylobacter* in the faeces of calves compared to dairy cows [36, 37].

In general, thermotolerant *Campylobacter*; mainly *C*. *jejuni* und *C*. *coli*, accounted for most human campylobacteriosis cases [80]. Nevertheless, other *Campylobacter* species such as *C*. *hyointestinalis* have also been reported to cause disease [81, 82]. It is important to mention that different methods of cultivation favour different species of *Campylobacter* [83]. *C*. *hyointestinalis* mainly colonized cows, but the cultural detection of *C*. *hyointestinalis* is not always ensured based on the fact that this species is not known to be thermotolerant and higher detection levels would occur after enrichment at 37°C compared with direct culture [84]. Still, the *Campylobacter* species *C*. *hyointestinalis* and *C*. *jejuni* are predominantly found in dairy cows [59, 62]. Accordingly, in the meta-analysis with the multilevel mixed-effect model *C*. *hyointestinalis* and *C*. *jejuni* had a significant impact on the pooled prevalence estimate (S4 Fig).

Repeated samplings are needed in order to examine whether the prevalence and concentration of *Campylobacter* in faeces of dairy cows follow a seasonal pattern. In total 14 studies have taken repeated samples according to season in temperate regions and made data available in their publication. Anyway, this were not enough data for subgroup analysis on the aggregated sample and only the multilevel mixed-effect model could be used to analyse the effect of seasons on the pooled prevalence estimate (S5 Fig). The results from the multilevel mixed-effect model showed no significant effect of seasons on the pooled prevalence estimate which was contrary to results reported by other studies [1, 34, 85]. Seasonal changes in *Campylobacter* concentration in cow faeces were expected based on the observations that the occurrence of *Campylobacter* in the faeces of food-producing animals has been shown to be subject to seasonal changes [3, 86] and that every year a seasonal increase in *Campylobacter* infections is recorded in the warmer months [85, 87, 88]. It has been shown that *Campylobacter* has a characteristic seasonality with a sharp increase of cases in the summer and a smaller but distinct winter peak [1]. Additionally, a distinct peak in the *Campylobacter* concentration in cow faeces in either winter or summer has been reported [89]. However, a bimodal trend with faecal extraction in spring and autumn has also been observed [34].

## Strengths and limitations of the study

This systematic review demonstrates the important data gaps for the meta-analysis of the prevalence and concentration of *Campylobacter* in cow’s faeces. The major hurdle in evaluating prevalence data for *Campylobacter* spp. in faeces of dairy cows from the literature was that the data were often made available only in an aggregated state (e.g. average per subgroup). Other identified data gaps were related to the missing metadata regarding the description of the population under study (e.g. age class and health status), the sampling conditions (e.g. season) or the methodology used (e.g. faecal collection method and the use of ISO methods for *Campylobacter* detection). Thus, meta-analysis and evaluation using the specific subgroups was significantly limited. A further limitation was based on the high heterogeneity between studies, which made an estimation of the prevalence difficult. This high heterogeneity was most likely based on the high degree of variability between studies in populations under study, sampling conditions, methodology and so on. In addition, heterogeneity was likely also affected by data aggregation and missing metadata.

Future studies should therefore consider publishing raw data in non-aggregated state in order to provide better re-usability of data and to move towards the Findability, Accessibility, Interoperability, and Reuse (FAIR) data principles for scientific data [90]. Moreover, we are suggesting that authors of future studies carefully consider which metadata to collect and report in their publications to further support re-usability.

In addition, we highlighted the importance of analysing the prevalence and concentration of *Campylobacter* in food-producing animals at farm levels in order to better understand and estimate potential cross-contamination mechanisms along the food chain. Specifically concentration data (including mean and standard deviation) are an important input for QMRAs and this review and meta-analysis emphasizes the need for more studies that collect concentration data for *Campylobacter* in dairy cow faeces.

Nevertheless, the analysis of the extracted prevalence data presented in this study is considered a valuable basis for the further development of QMRAs and different risk mitigation strategies along the raw milk supply chain for *Campylobacter* spp. in (raw) milk and food products thereof.

## Supporting information

S1 Checklist PRISMA checklist. (DOC)

S1 Fig Heterogeneity. (A) Heterogeneity in the multilevel model, (B) Heterogeneity in the multilevel mixed effects model.
(TIF)

S2 Fig Bubbleplot. (TIF)

S3 Fig Forest plot RoB. (TIF)

S4 Fig Forest plot species. (TIF)

S5 Fig Forest plot season. (TIF)

S1 Table Extraction table. (XLSX)

S2 Table Risk of bias. (XLSX)
