# Supplementary figures and images for: Prevalence and concentration of Campylobacter in faeces of dairy cows: A systematic review and meta-analysis
Source: PLoS One. 2022 Oct 14;17(10):e0276018. doi: 10.1371/journal.pone.0276018 (PMC9565387; doi:10.1371/journal.pone.0276018)

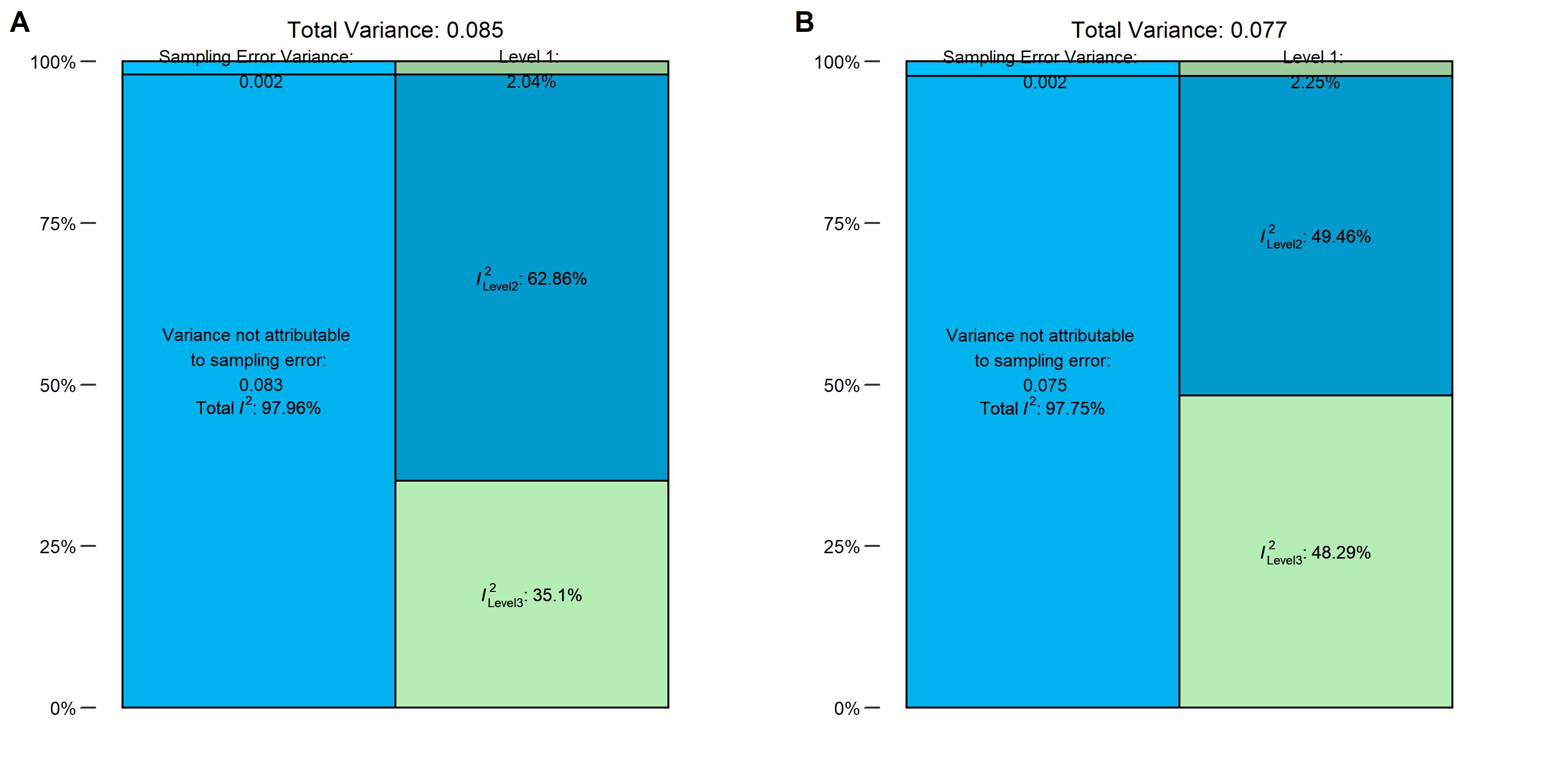

Supplement: S1 Fig — (A) Heterogeneity in the multilevel model, (B) Heterogeneity in the multilevel mixed effects model. (TIF) [file pone.0276018.s002.tif]

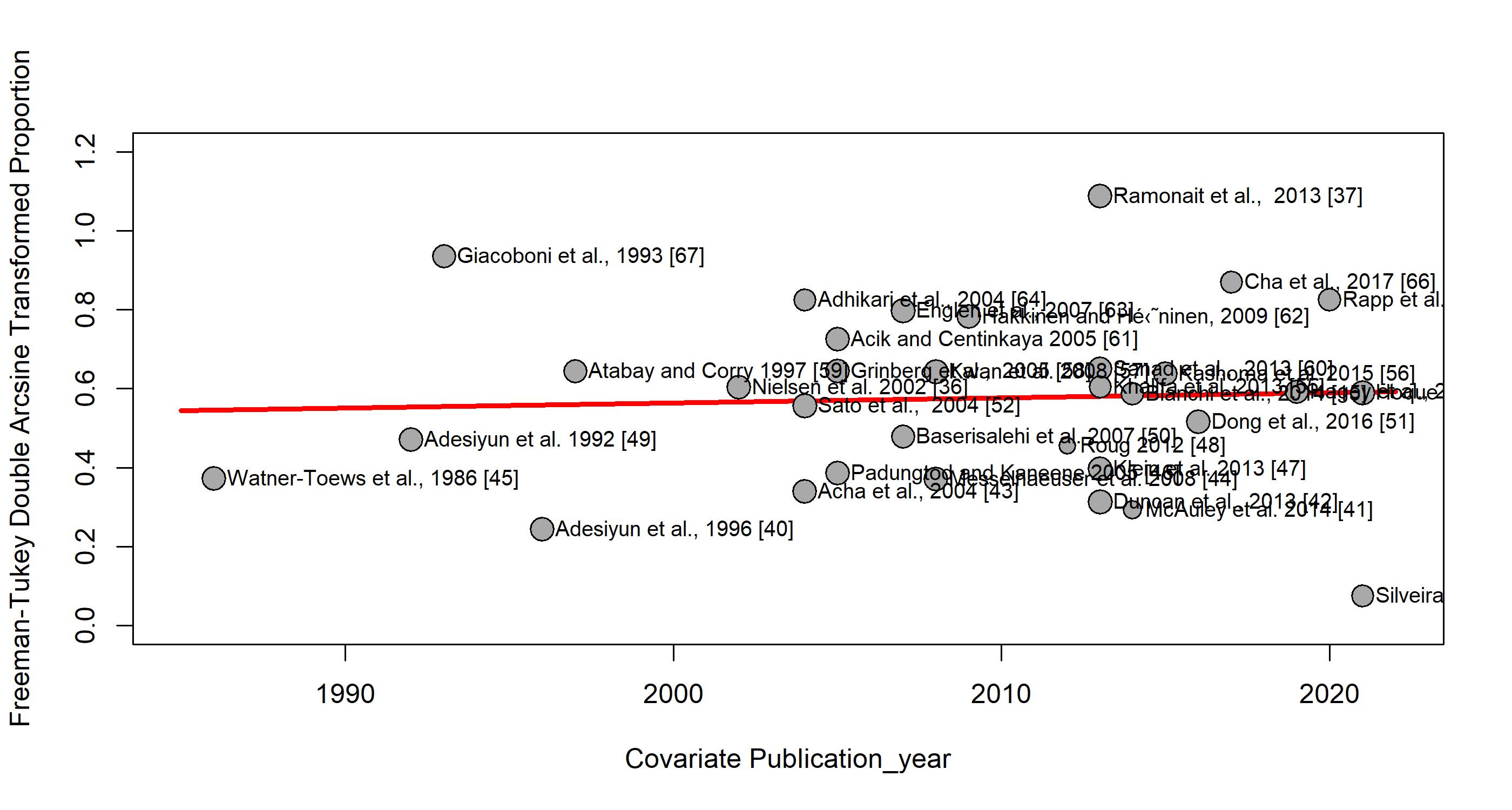

Supplement: S2 Fig — (TIF) [file pone.0276018.s003.tif]

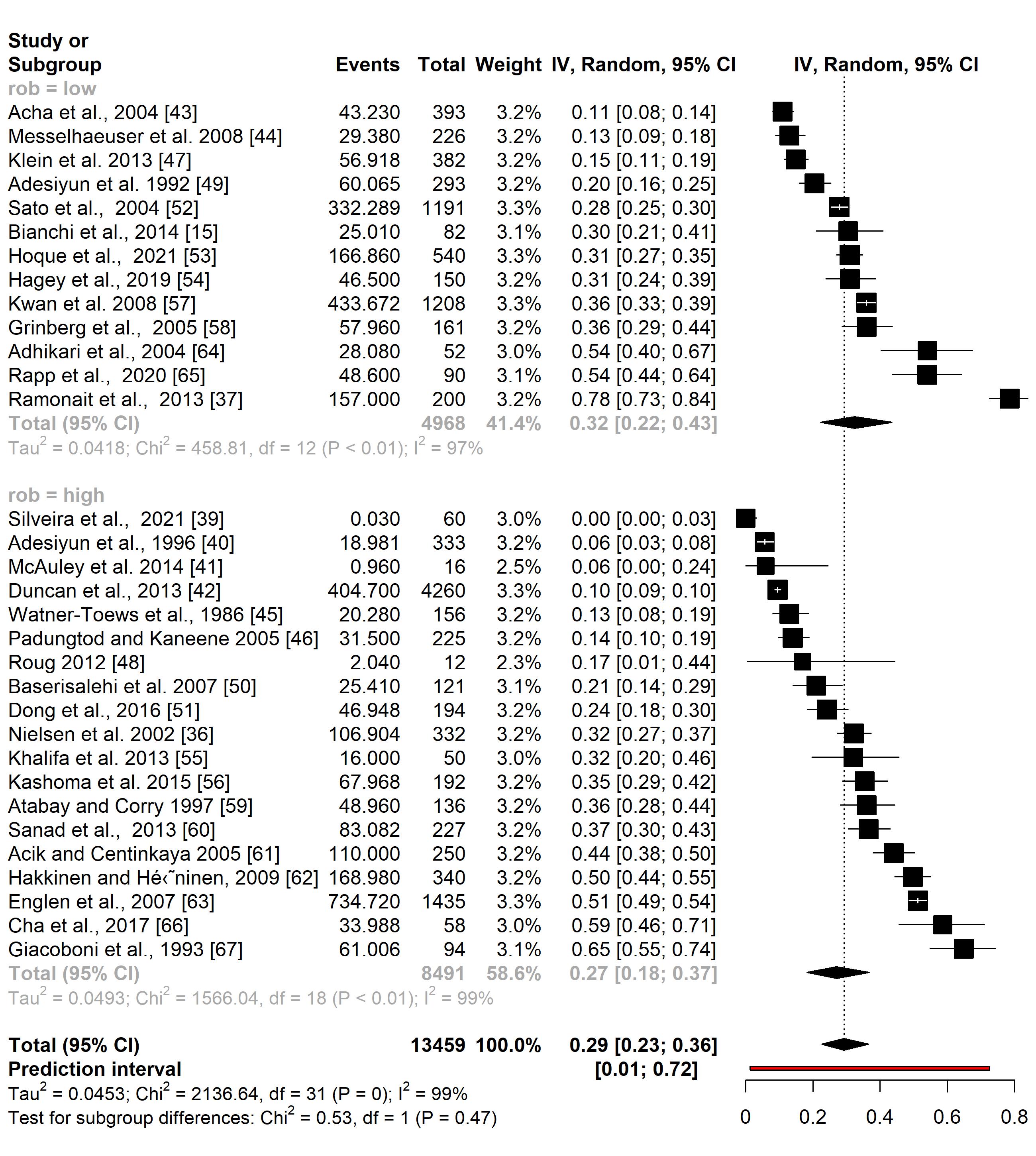

Supplement: S3 Fig — (TIF) [file pone.0276018.s004.tif]

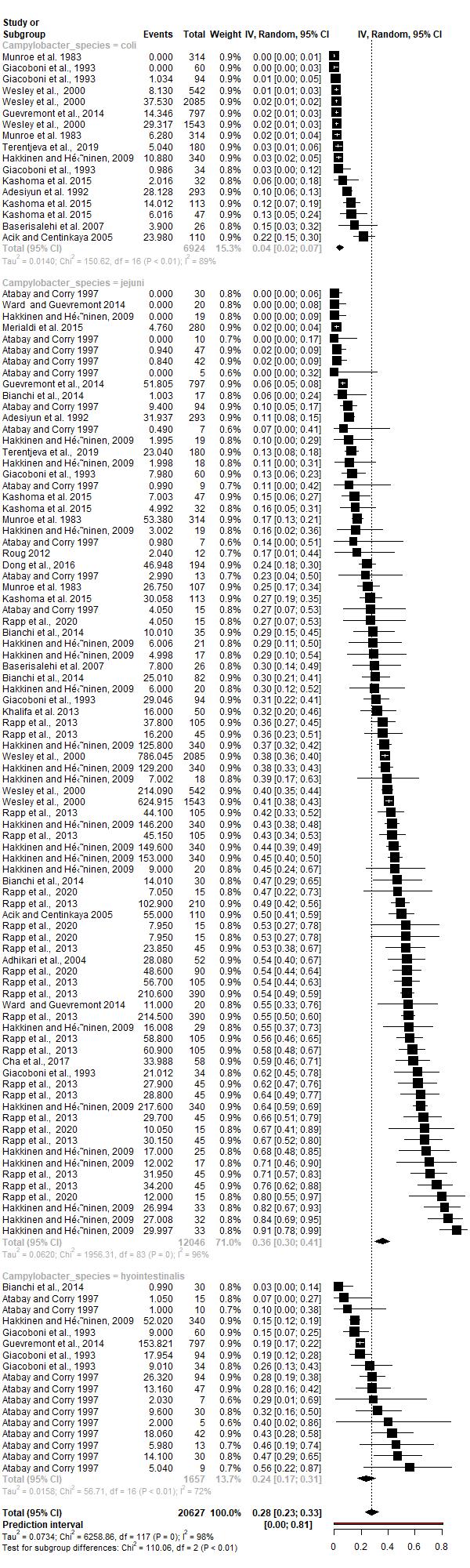

Supplement: S4 Fig — (TIF) [file pone.0276018.s005.tif]

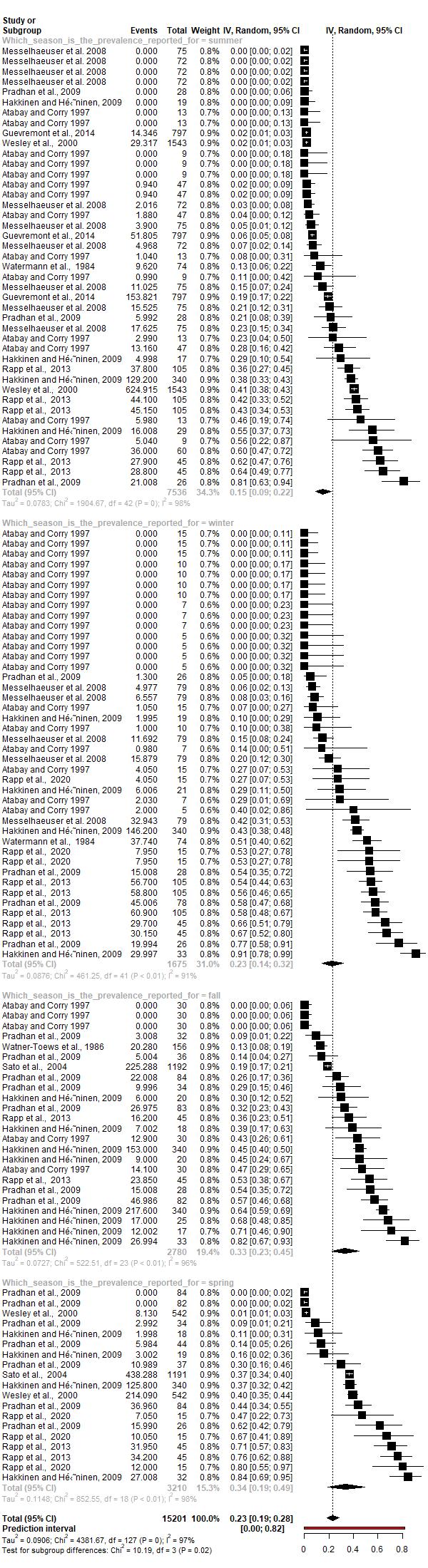

Supplement: S5 Fig — (TIF) [file pone.0276018.s006.tif]
